# Supplementary material for: Evolving Role of RING1 and YY1 Binding Protein in the Regulation of Germ-Cell-Specific Transcription
Source: Genes (Basel). 2019 Nov 19;10(11):941. doi: 10.3390/genes10110941 (PMC6895862; doi:10.3390/genes10110941)
Supplement: Supplementary file 1 [file genes-10-00941-s001.pdf]

Supplementary data 1:

List of 65 commonly upregulated genes from the three *Rybp* transcriptome available from ES cells sources- (Ujhelly et al., 2015, Rose et al.,2016 and Zhao et al., 2018). The genes are listed in the descending order of their log2foldchange values as per Ujhelly et al., 2015.

1. *Calb2*
2. *Slc25a31*
3. *Stk31*
4. *Adcy7*
5. *Syce1*
6. *Chrna4*
7. *Ctcfl*
8. *Aox3*
9. *1700016K19Rik*
10. *Nxf2*
11. *Gatm*
12. *4930502E18Rik*
13. *Dmrtb1*
14. *Sycp2*
15. *Fam26f*
16. *Fbp2*
17. *Ildr2*
18. *Apba1*
19. *Slc4a1*
20. *Myh13*
21. *Egfl6*
22. *Plekhg4*
23. *Btn2a2*
24. *Vav1*
25. *Pogk*
26. *Cygb*
27. *Rftn1*
28. *Mael*
29. *Taf7l*
30. *Ikbke*
31. *Iqsec3*
32. *Dazl*
33. *Tbx3*
34. *Cdh4*
35. *Bsn*
36. *Ky*
37. *Hpcal4*
38. *Tns1*
39. *Car6*
40. *Ddr2*
41. *Atp2b4*
42. *Ddx4*
43. *Tdrkh*
44. *Trpa1*
45. *Brdt*
46. *Pramel1*
47. *Rcsd1*
48. *Ptpre*
49. *Piwil2*
50. *Boll*
51. *Dram1*
52. *Hsf5*
53. *Crabp1*
54. *Rnf219*
55. *Tex11*
56. *Ido2*
57. *Tex101*
58. *Mlf1*
59. *Tdrd1*
60. *Dgat2*
61. *Arsb*
62. *P2rx5*
63. *Sh2d4b*
64. *Pde3a*
65. *Scml4*

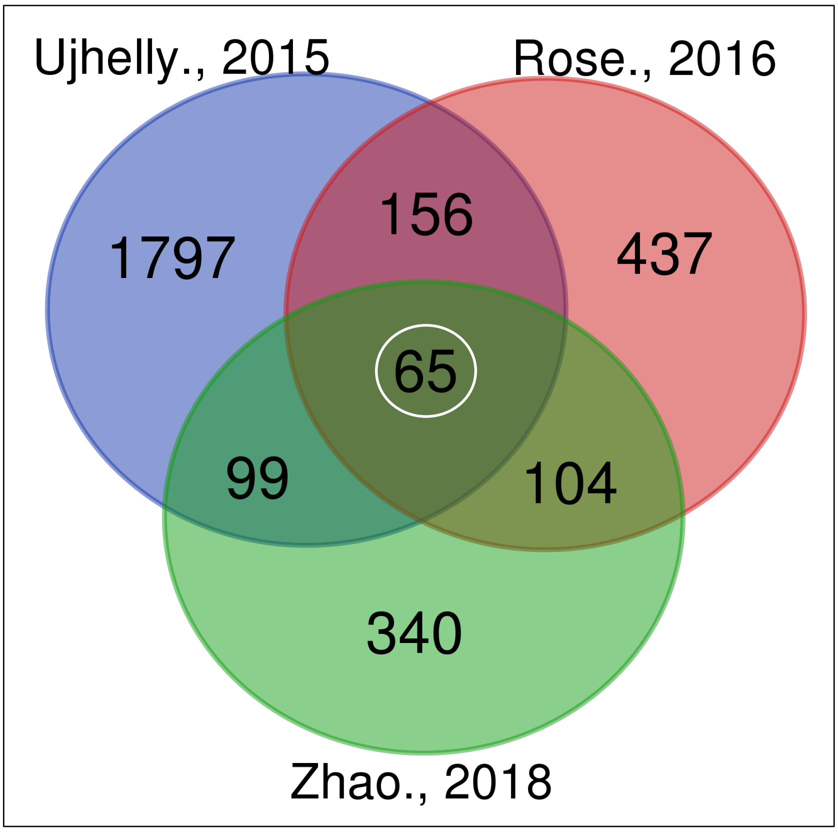
Ujhelly., 2015
Rose., 2016
Zhao., 2018
1797
156
437
65
99
104
340

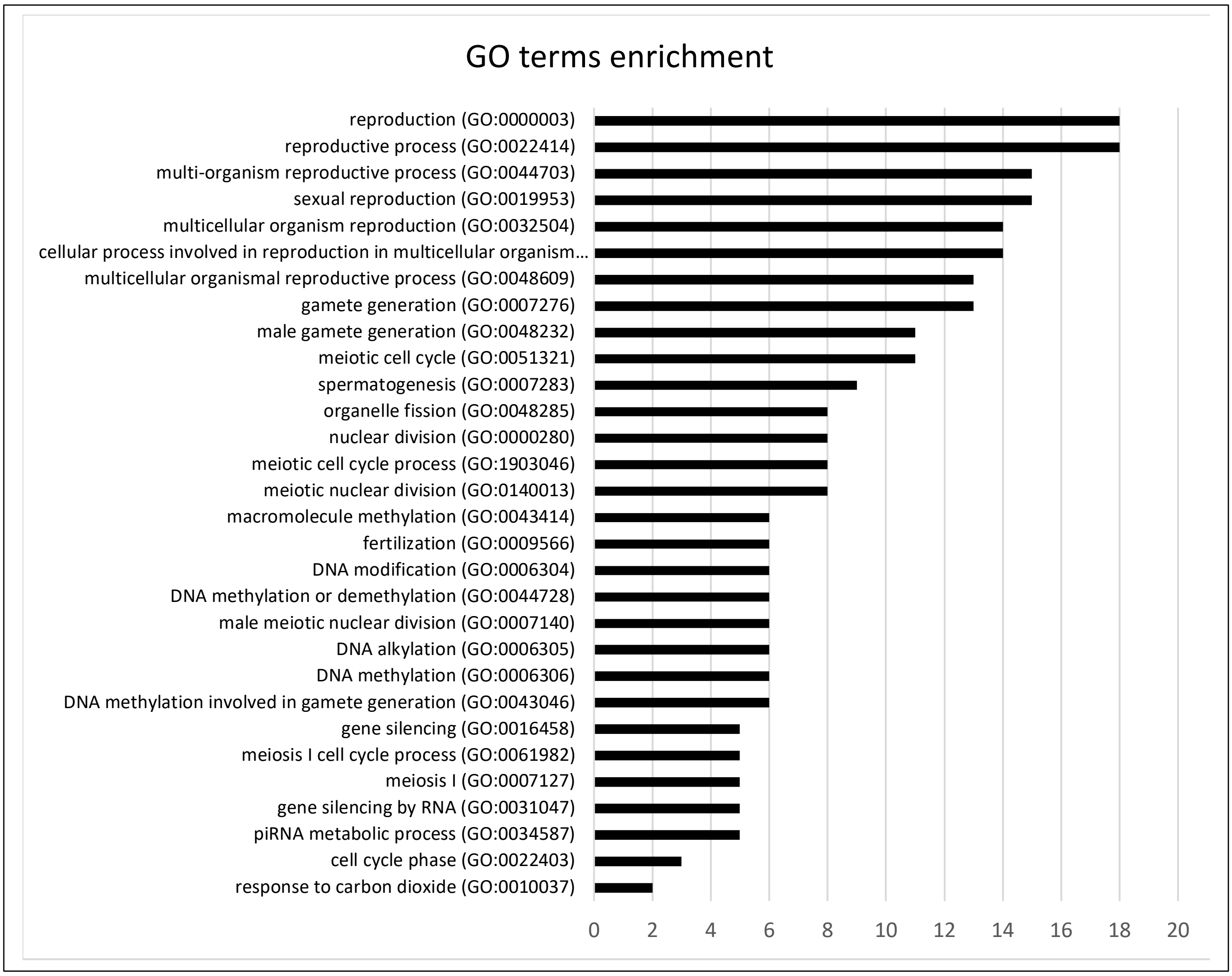
GO terms enrichment
reproduction (GO:0000003)
reproductive process (GO:0022414)
multi-organism reproductive process (GO:0044703)
sexual reproduction (GO:0019953)
multicellular organism reproduction (GO:0032504)
cellular process involved in reproduction in multicellular organism...
multicellular organismal reproductive process (GO:0048609)
gamete generation (GO:0007276)
male gamete generation (GO:0048232)
meiotic cell cycle (GO:0051321)
spermatogenesis (GO:0007283)
organelle fission (GO:0048285)
nuclear division (GO:0000280)
meiotic cell cycle process (GO:1903046)
meiotic nuclear division (GO:0140013)
macromolecule methylation (GO:0043414)
fertilization (GO:0009566)
DNA modification (GO:0006304)
DNA methylation or demethylation (GO:0044728)
male meiotic nuclear division (GO:0007140)
DNA alkylation (GO:0006305)
DNA methylation (GO:0006306)
DNA methylation involved in gamete generation (GO:0043046)
gene silencing (GO:0016458)
meiosis I cell cycle process (GO:0061982)
meiosis I (GO:0007127)
gene silencing by RNA (GO:0031047)
piRNA metabolic process (GO:0034587)
cell cycle phase (GO:0022403)
response to carbon dioxide (GO:0010037)
0
2
4
6
8
10
12
14
16
18
20

Supplementary data 2:

Gene Ontology (GO) analysis of the 65 commonly upregulated genes identified with germ cell, meiotic and spermatogenic functions.

Germ cell-

1. *Tex11*
2. *Syce1*
3. *Mael*
4. *Atp2b4*
5. *Tdrkh*
6. *Boll*
7. *Piwil2*
8. *1700016K19Rik*
9. *Brdt*
10. *Tex101*
11. *Pde3a*
12. *Ctcfl*
13. *Tbx3*
14. *Tdrd1*
15. *Taf7l*
16. *Ddx4*
17. *Sycp2*
18. *Dazl*

Meiotic-

1. *Tex11*
2. *Syce1*
3. *Mael*
4. *Tdrkh*
5. *Boll*
6. *Piwil2*
7. *Brdt*
8. *Tdrd1*
9. *Ddx4*
10. *Sycp2*
11. *Dazl*

Spermatogenesis-

1. *Mael*
2. *Tdrkh*
3. *Boll*
4. *Piwil2*
5. *Brdt*
6. *Tdrd1*
7. *Taf7l*
8. *Ddx4*
9. *Dazl*

Supplementary data 3:

<u>Common target analysis</u>

Finding common genes amongst the 2117 significantly (more than 2-fold) upregulated genes in *Rybp*$^{-/-}$ mouse ES cells (Ujhelly et al., 2015) and 1481 NANOG bound genes in human ES cells (Boyer et al., 2005)

List of 81 common hits

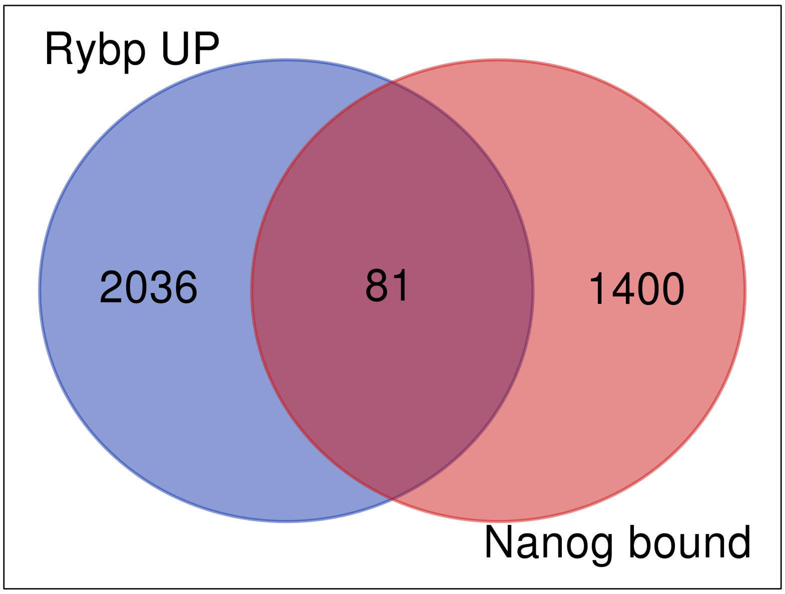

1. *Tsnaxip1*
2. *Tle2*
3. *Ppp1r12b*
4. *Slc30a1*
5. *Ascl2*
6. *Sfrp1*
7. *Rab17*
8. *Igfbp3*
9. *Mmp9*
10. *Hoxc4*
11. *Irx2*
12. *Nadsyn1*
13. *Hectd2*
14. *Pxmp3*
15. *Cyp2r1*
16. *Zcchc12*
17. *Dmrt1*
18. *Polr3d*
19. *Csad*
20. *Rgs9*
21. *Ly96*
22. *Susd1*
23. *Anxa8*
24. *Fam54a*
25. *Tmem38a*
26. *Amigo2*
27. *Naalad2*
28. *Rgs20*
29. *Adamtsl1*
30. *Prnp*
31. *Cerkl*
32. *Apoa2*
33. *Pbx1*
34. *Kbtbd10*
35. *Serpinh1*

36. *Ddx49*
37. *Slc13a4*
38. *Hoxd1*
39. *Kif9*
40. *Pde10a*
41. *Epm2aip1*
42. *Casp9*
43. *Olfml3*
44. *Calcrl*
45. *Pax3*
46. *Pmaip1*
47. *Sfi1*
48. *Eomes*
49. *Nr3c2*
50. *Phyhip*
51. *Klhl5*
52. *Tmc1*
53. *Rnf17*
54. *Psen2*
55. *Hoxb9*
56. *Rbm24*
57. *Lrp3*
58. *Lhx2*
59. *Usf1*
60. *Gad2*
61. *Ier5l*
62. *Gga1*
63. *Cttnbp2*
64. *Spaca1*
65. *Rgs10*
66. *Chrne*
67. *Kcnn2*
68. *Ppp1r16b*
69. *Fkbp7*
70. *Bmp7*
71. *Tpcn2*
72. *Hesx1*
73. *Asb1*
74. *Gng12*
75. *Pygm*
76. *Igfbp2*
77. *Rab38*
78. *Hist1h3i*
79. *C1qtnf6*
80. *Lhpp*
81. *Pik3r2*
